# BCAR3 promotes head and neck cancer growth and is associated with poor prognosis

**DOI:** 10.1038/s41420-021-00714-7

**Published:** 2021-10-27

**Authors:** Ze Zhang, Yafei Wang, Yun Wang, Chunli Wang, Yanjie Shuai, Jingtao Luo, Ruoyan Liu

**Affiliations:** 1 Department of Maxillofacial and Otorhinolaryngology Oncology, and Department of Head and Neck Oncology, Tianjin Medical University Cancer Institute and Hospital, Tianjin, 300060 China; 2 Key Laboratory of Cancer Prevention and Therapy, National Clinical Research Center for Cancer, Tianjin, 300060 China; 3 Tianjin’s Clinical Research Center for Cancer, Tianjin, 300060 China; 4 Department of Gynaecological Oncology, Tianjin Medical University Cancer Institute and Hospital, Tianjin, 300060 China

**Keywords:** Head and neck cancer, Prognostic markers

## Abstract

Breast cancer anti-estrogen resistance protein 3 (BCAR3) is involved in anti-estrogen resistance and other important aspects of breast cancer. However, the role of BCAR3 in other solid tumors remains unclear. The relationship between the clinicopathologic characteristics of head and neck squamous cell carcinoma (HNSCC) patients and BCAR3 was analyzed using the Wilcoxon’s signed-rank test and logistic regression. The association between BCAR3 expression and clinicopathologic features and survival was analyzed using Cox regression and the Kaplan–Meier method. In vivo and in vitro assays were performed to validate the effect of BCAR3 on HNSCC growth. BCAR3-related mRNAs were determined by calculating the Pearson’s correlation coefficient based on The Cancer Genome Atlas (TCGA). Gene Ontology (GO) and Kyoto Encyclopedia of Genes and Genomes (KEGG) pathway enrichment analyses, and gene set enrichment analysis (GSEA) were used to predict the potential functions of BCAR3. BCAR3 expression is overexpressed in HNSCC and was shown to be associated with perineural invasion (PNI) and poor survival. BCAR3 silencing significantly attenuated the proliferation of HNSCC cells, whereas BCAR3 depletion inhibited tumor growth in vitro. GO and KEGG functional enrichment analyses, and GSEA showed that BCAR3 expression in HNSCC was associated with biological processes, such as cell adhesion, actin binding, cadherin binding, and angiogenesis. BCAR3, which promotes HNSCC growth, is associated with perineural invasion and may be a potential molecular prognostic marker of poor survival in HNSCC.

## Introduction

Head and neck squamous cell carcinoma (HNSCC), which is derived from the mucosal epithelium in the oral cavity, pharynx, and larynx, is the sixth most common cancer, with ~890,000 new cases and 450,000 deaths per year worldwide [1, 2]. Tobacco consumption, alcohol abuse, or both, and infection with human papillomavirus (HPV) are recognized as the main causes of HNSCC [2, 3]. Most HNSCC patients are treated with surgery, chemotherapy, radiotherapy, and molecular targeted therapy. Although several targeted agents, such as cetuximab (therapeutic antibody that targets epidermal growth factor (EGF) receptor) and pembrolizumab or nivolumab (programmed cell death protein 1 inhibitors), have been approved by the US Food and Drug Administration for HNSCC treatment, overall response rates have been moderate [2]. Moreover, the survival of HNSCC patients has improved only modestly over the past few decades according to data within the Surveillance, Epidemiology, and End Results registry [4]. Therefore, unraveling the molecular mechanisms of HNSCC and identifying prognostic biomarkers are still critical, and may provide disease-specific opportunities for therapeutic exploitation.

Breast cancer anti-estrogen resistance protein 3 (BCAR3), also known as NSP2, was initially identified in a study in which genes involved in the anti-estrogen resistance of breast cancer cells were determined; in that study, several genes were collectively named breast cancer anti-estrogen resistance (BCAR) genes [5]. Subsequently, BCAR3 was found to interact with BCAR1 (p130^Cas^) and play a role in the activation of Rac, Cdc42, and Cyclin D1 promoter, as well as in the regulation of cell migration and cellular response to EGF stimulation [6, 7]. On the clinical level, BCAR3 was found to be associated with the survival and prognosis of patients with breast cancer [8, 9]. It is worth noting that existing studies on BCAR3 have mainly focused on its molecular mechanism and clinical significance in breast cancer. Thus far, studies concerning BCAR3 in other types of cancer remain scarce and the role of BCAR3 in HNSCC is currently unknown.

In the present study, we found that BCAR3 expression is significantly associated with poor clinical outcomes of HNSCC cancer patients. BCAR3 silencing suppresses HNSCC growth both in vivo and in vitro. BCAR3-related mRNAs were analyzed with Gene Ontology (GO) and Kyoto Encyclopedia of Genes and Genomes (KEGG) analyses, and the results showed that BCAR3 is likely associated with many important biological functions, such focal adhesion and actin regulation. Gene set enrichment analysis (GSEA) showed that epithelial mesenchymal transition (EMT), angiogenesis, apical junctions, inflammatory responses, and focal adhesion were associated with a BCAR3 high-expression phenotype.

## Materials and methods

### RNA-sequencing data download and bioinformatics analysis

The gene expression data (500 cases) and corresponding clinical information were downloaded from The Cancer Genome Atlas (TCGA) official website for HNSCC. To evaluate the prognostic effects of BCAR3 expression and other clinical factors, data were used from 499 patients with an available survival status. Data were obtained from 44 patients with matched cancer and normal tissues, which were used to assess BCAR3 mRNA expression level differences between HNSCC tissues and adjacent normal tissues. An oral carcinoma mRNA microarray profiling data set (GSE31056) was obtained from the GEO database (https://www.ncbi.nlm.nih.gov/geo/query/acc.cgi?acc=GSE31056). Twenty-two oral carcinoma tissues and 24 normal tissues were analyzed in this array (platform: GPL10526 [HG-U133_Plus_2] Affymetrix GeneChip Human Genome HG-U133 Plus 2 Array).

### Cell culture

Human HNSCC cells (SCC25 and FaDu) were maintained in Dulbecco’s modified Eagle’s medium (DMEM)/F12 medium (HyClone, USA) supplemented with 10% fetal bovine serum (Gibco, USA) and 2 mM glutamine in a 5% CO_2_ atmosphere at 37 °C.

### siRNA transfection and lentiviral infection

Specific or scrambled short interfering RNAs (RiboBio, China) were used to transfect HNSCC cells using Lipofectamine 2000 (Invitrogen, USA). The lentiviral delivery system of short hairpin RNAs (shRNAs) and scramble vector (-hPGK-Puro-CMV-tGFP) used for the in vivo and in vitro experiments was purchased from Sigma (shRNA, Sigma-Aldrich, USA). Puromycin (2 µg/ml; Sigma-Aldrich, USA) was used to select infected cells for 1 week. The efficiency of silencing was assessed by western blotting.

### Western blotting

Cells were collected and lysed in lysis buffer for 30 min at 4 °C and total protein was quantified using a BCA protein assay kit (Thermo Fisher Scientific, USA). The proteins were dissociated and separated by SDS-polyacrylamide gel electrophoresis and then transferred to polyvinylidene difluoride membranes, which were incubated with primary antibodies at 4 °C. The primary antibodies used for western blotting and their sources were as follows: anti-BCAR3 (Atlas, HPA014858, Sweden) and anti-GAPDH (Cell Signaling Technology, #5174, USA). Antigen–antibody complexes were detected using horseradish peroxidase-conjugated secondary antibodies (Cell Signaling Technology, #7074; #7076, USA) with enhanced chemiluminescence western blotting detection reagent (Merck Millipore, USA).

### MTT cell proliferation assay

Cell survival and proliferation assays were performed using the methyl-thiazolyl diphenyl-tetrazolium bromide (MTT) method. After cells were seeded into 96-well plates, a total volume of 20 μl of MTT solution (Solarbio, China, 5 mg/ml) was added and incubated for 4 h at 37 °C in the dark. After the medium and MTT were removed, 200 μl of dimethyl sulfoxide was added to each well and the optical density at an absorbance wavelength of 490 nm was measured by spectrophotometry (NanoDrop™ 3300, Thermo Scientific™, USA).

### EdU cell proliferation assay

HNSCC cells were fixed in 4% paraformaldehyde in phosphate-buffered saline (PBS) for 10 min, after which permeabilization buffer (0.5% Triton X-100 in PBS) was added. Then, 100 μl of 1×Apollo 643 (RiboBio, C10310-2, China) was added at which point the cells were incubated in the dark for 30 min followed by incubation with 4′,6-diamidino-2-phenylindole for 30 min. Images were acquired with a fluorescence microscope (IN Cell Analyzer 6500HS, GE, USA).

### In vivo tumor xenograft model

BALB/cA-nu female nude mice (5–6 weeks of age) were randomly divided into groups (seven mice per group) and cells were injected subcutaneously into their right flanks. Infected cells from stable, single cell clones of shBCAR3 or control cells (4 × 10^6^ cells in 100 μl serum-free DMEM) were injected into each nude mouse. The National Institutes of Health Guide for the Care and Use of Laboratory Animals was followed in this study.

### Gene set enrichment analysis

GSEA analysis was performed using GSEA 4.1.0 (http://www.broadinstitute.org/gsea/). Of the 500 HNSCC patients, the 125 with the highest BCAR3 expression (top 25%) and 125 with the lowest BCAR3 expression (bottom 25%) were divided into two groups. A nominal *p*-value < 0.05 and a false discovery rate (FDR) *q*-value < 0.25 were used as cutoffs for significance. MSigDB collections of h.all.v7.4.symbols[Hallmarks] and c2.cp.kegg.v7.4.symbols[Curated] were analyzed.

### Identification of BCAR3-related mRNAs and GO analyses

The coexpression relationships of BCAR3 mRNA and other mRNAs were determined by calculating the Pearson’s correlation coefficients. If the absolute values of the Pearson’s correlation coefficients between the other mRNAs and BCAR3 mRNA were >0.3 and if the *p*-value < 0.01, the mRNAs were considered BCAR3-related mRNAs. The “clusterprofiler” R package was used to perform GO and KEGG pathway analyses.

### Cell migration and invasion assays

Transwell cell culture chambers were used for cell migration and invasion assays. Upper membranes were precoated with Matrigel as a barrier for the invasion assay. A total of 6 × 10^4^ cells were placed into the upper chamber. After 24 h, the cells in the chambers were fixed in 4% paraformaldehyde for 30 min and then stained. Cells that migrated toward the outer chamber were counted.

### Statistical analysis

R version 4.0.3 was used for the statistical analyses. The relationship between the clinical pathologic features of HNSCC and BCAR3 was analyzed using the Wilcoxon’s signed-rank test and logistic regression. Whether clinicopathologic characteristics and BCAR3 expression were associated with overall survival in patients from the TCGA database was determined using Cox regression and the Kaplan–Meier method. Hazard ratios were calculated using a Cox proportional hazards model with the “survminer” R package.

## Results

### Association of BCAR3 expression with survival and other clinicopathologic variables

**Fig. 1:**
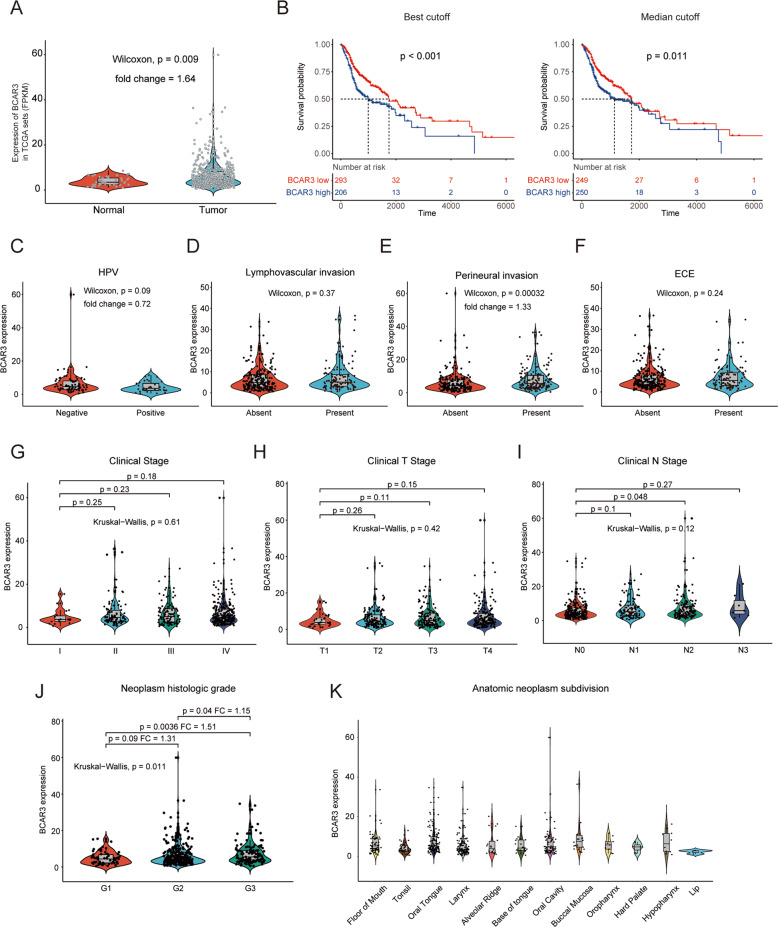
BCAR3 is upregulated in HNSCC and increased BCAR3 expression is correlated with poor prognosis in patients with HNSCC. **A** The expression level of BCAR3 was upregulated in HNSCC tissues (*n* = 500) compared with adjacent normal tissues (*n* = 44) in the TCGA cohort. **B** Kaplan–Meier analysis results for the overall survival correlation with BCAR3 expression, as assessed by sequencing in the TCGA cohort, are shown. Left panel: patients in the TCGA cohort were divided into two groups according to the cutoff representing the value that yields the maximal difference. Right panel: patients were divided into two groups according to the median value of BCAR3 expression. Clinicopathologic characteristics associated with BCAR3 expression including **C** HPV, **D** lymphovascular invasion, **E** perineural invasion, **F** ECE, **G** clinical stage, **H** clinical T stage, **I** clinical N stage, **J** neoplasm histologic grade, and **K** anatomic neoplastic subdivision are shown. Note that each dot represents one patient. ECE, extracapsular extension; FC, fold change; HNSCC, head and neck squamous cell carcinoma; HPV, human papillomavirus; TCGA, The Cancer Genome Atlas.

To investigate the role of BCAR3 in HNSCC, we first conducted an analysis to evaluate the mRNA expression level difference between HNSCC tissues and adjacent normal tissues in the TCGA cohort, and found that BCAR3 was significantly overexpressed in HNSCC (Fig. 1A). Similarly, overexpression of BCAR3 was also found in tumor tissues based on data from another cohort from the GEO database (GSE31056) (Supplementary Fig. 1A). Next, we investigated the prognostic significance of BCAR3 in HNSCC. The patient group with high BCAR3 expression exhibited worse overall survival when patients were divided according to the median expression value. The difference was more significant when patients were divided according to the cutoff representing the value that yielded the maximal difference (Fig. 1B). We further evaluated the association between BCAR3 expression and clinicopathologic variables. As shown in Fig. 1C–J, increased BCAR3 expression was significantly correlated with a worse differentiation grade and an increased probability of perineural invasion. Patients with HPV-negative status exhibited a trend toward higher BCAR3 expression compared with HPV-positive patients, although the difference was not significant. A univariate analysis using logistic regression also revealed that BCAR3 expression as a categorical dependent variable (based on the median value) was associated with perineural invasion (odds ratio (OR) = 1.720, absent vs. present) (Table 1). Notably, BCAR3 expression was associated with administration of lymph nodes neck dissection (OR = 1.934, none vs. done) (Table 1) and was inversely associated with administration of radiotherapy (OR = 0.660, none vs. done) (Table 1). To reveal whether BCAR3 expression and clinicopathologic variables are associated with poor survival in HNSCC, we first performed a univariate analysis, which revealed that BCAR3 expression was significantly correlated with poor overall survival (Table 2). Other clinicopathologic variables associated with poor survival included older age, female sex, lymphovascular invasion, extracapsular extension, perineural invasion, new tumor after initial treatment, positive margins, absence of radiotherapy, and number of positive lymph nodes (Table 2). No significant difference was observed in BCAR3 expression among anatomic neoplastic subdivisions of the floor of the mouth, tonsil, oral tongue, larynx, alveolar ridge, base of the tongue, oral cavity, buccal mucosa, oropharynx, hard palate, hypopharynx, and lip (Fig. 1K).

**Table 1:** BCAR3 expression^a^ associated with clinical pathological characteristics (logistic regression).

Clinicopathologic variable	Odds ratio in BCAR3 expression	95% Confidence interval	*P*-value
Age (continuous)	0.993	0.978–1.008	0.336
Gender (female vs. male)	0.831	0.558–1.237	0.363
Alcohol history (no vs. yes)	1.052	0.719–1.538	0.795
Smoke history (no vs. yes)	0.966	0.706–1.319	0.827
HPV (negative vs. positive)	0.488	0.203–1.120	0.097
Histologic grade (G1 vs. G2 vs. G3)	1.218	0.905–1.644	0.195
Lymphovascular invasion (absent vs. present)	1.183	0.756–1.855	0.463
ECE (absent vs. present)	1.140	0.725–1.800	0.571
Perineural invasion (absent vs. present)	1.720	1.126–2.639	**0.012**
New tumor after initial treatment (absent vs. present)	1.250	0.649–2.433	0.506
Margin (negative vs. positive)	1.081	0.699–1.676	0.725
Lymphnode neck dissection (none vs. done)	1.934	1.215–3.123	**0.006**
Radiotherapy (none vs. done)	0.660	0.444–0.980	**0.040**
Molecular targeted therapy (none vs. done)	0.899	0.595–1.357	0.613
Number of positive lymph nodes (continuous)	1.013	0.961–1.072	0.636
Clinical stage (I or II vs. III or IV)	1.215	0.798–1.853	0.365
Clinical N stage (cN0 vs. cN+)	1.352	0.944–1.940	0.100
Clinical T stage (T1 or T2 vs. T3 or T4)	1.219	0.842–1.768	0.295

^a^Categorical dependent variable, greater or less than the median expression level.

The bold values mean statistical significance.

**Table 2:** Univariate Cox regression analysis of various prognostic parameters in the TCGA patients.

Clinicopathologic variable	Total (*N*)	Hazard ratio	95% Confidence interval	*P*-value
Age (continuous)	499	1.020	1.007–1.032	**0.002**
Gender (female vs. male)	499	0.7428	0.5583–0.9882	**0.040**
Alcohol history (no vs. yes)	488	0.9741	0.7321–1.296	0.857
Smoke history (no vs. yes)	489	0.7784	0.5971–1.015	0.066
HPV (negative vs. positive)	112	0.5178	0.1971–1.36	0.174
Histologic grade (G1 vs. G2 vs. G3)	480	1.112	0.9005–1.373	0.324
Lymphovascular invasion (absent vs. present)	338	1.716	1.223–2.407	**0.002**
ECE (absent vs. present)	346	2.655	1.916–3.678	**<0.001**
Perineural invasion (absent vs. present)	350	2.210	1.563–3.124	**<0.001**
New tumor after initial treatment (absent vs. present)	189	3.433	2.091–5.636	**<0.001**
Margin (negative vs. positive)	447	1.592	1.168–2.169	**0.003**
Lymphnode neck dissection (none vs. done)	496	0.732	0.5267–1.017	0.062
Radiotherapy (none vs. done)	438	0.629	0.4636–0.8536	**0.003**
Molecular targeted therapy (none vs. done)	398	0.877	0.6149–1.252	0.470
Number of positive lymph nodes (continuous)	391	1.029	1.006–1.052	**0.011**
Clinical stage (I or II vs. III or IV)	484	1.239	0.9282–1.653	0.145
Clinical N stage (cN0 vs. cN+)	477	1.268	0.9688–1.661	0.083
Clinical T stage (T1 or T2 vs. T3 or T4)	485	1.221	0.8807–1.692	0.230
BCAR3 FPKM value (continuous)	499	1.02	1.002–1.038	**0.028**
BCAR3 group divided by median (low vs. high)	499	1.414	1.081–1.85	**0.011**
BCAR3 group divided by best cutoff (low vs. high)	499	1.575	1.204–2.06	**<0.001**

The bold values mean statistical significance.

### Role of BCAR3 in HNSCC

**Fig. 2:**
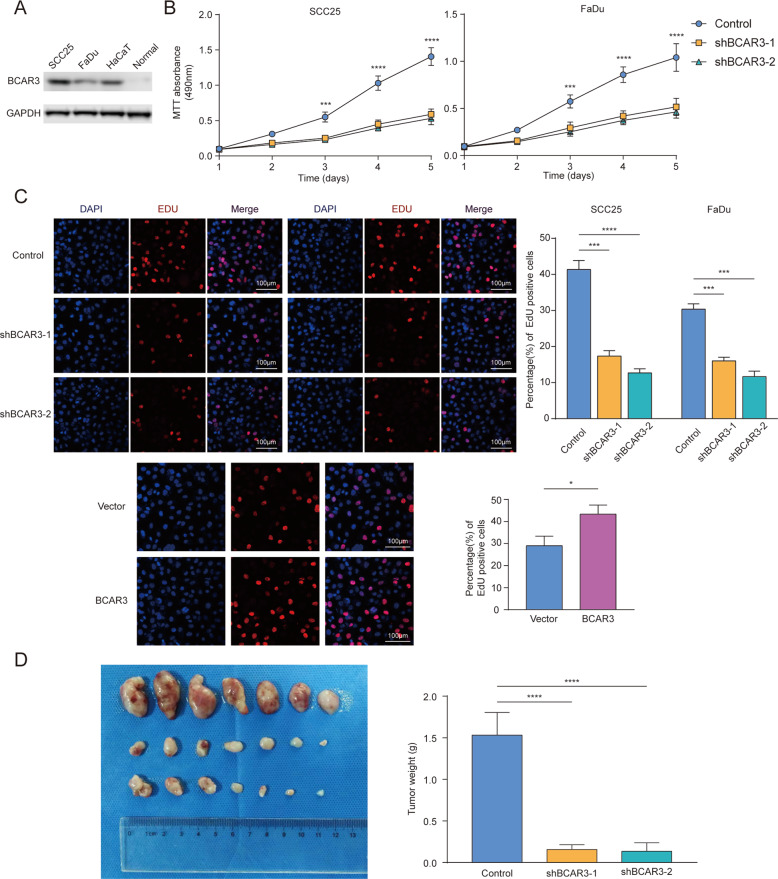
BCAR3 promotes HNSCC growth both in vitro and in vivo. **A** Immunoblotting analysis of BCAR3 protein levels in HNSCC cell lines (SCC25 and FaDu), a control cell line (HaCaT), and normal tongue tissue. **B** Proliferation of SCC25 and FaDu cells in which BCAR3 was silenced was evaluated by MTT assays. The experiment was repeated three times. Error bars indicate SD. **C** An EdU assay was performed to evaluate the proliferation of HNSCC with and without BCAR3 silencing or overexpression. The experiment was repeated three times. Error bars indicate SD. The number of EdU-positive cells per 100 cells was counted. **D** SCC25 cells with and without targeted BCAR3 interference were implanted subcutaneously into the right flanks of nude mice (6 × 100^6^ cells/mouse in 100 ml of serum-free medium). Tumor weights were measured in xenograft mice in the shBCAR3 and negative control groups. “control” represents “scramble vector.” *p < 0.05, **p < 0.01, ***p < 0.001, and ****p < 0.0001, respectively.

We first explored BCAR3 expression in SCC25, FaDu, HaCaT, and normal adjacent tissue obtained during oral cancer surgeries. To illustrate the effect of BCAR3 on HNSCC cell proliferation, we silenced BCAR3 in SCC25 and FaDu cells using two different shRNAs (shBCAR3-1 and shBCAR3-2); BCAR3 silencing was then verified by western blot analysis (Supplementary Fig. 2). In the MTT assay, the cell growth curves showed that BCAR3 silencing significantly attenuated the proliferation of both SCC25 and FaDu cells (Fig. 1A). Similarly, in the EdU assay, HNSCC cells with BCAR3 interference displayed a reduced percentage of EdU-positive cells compared with the negative control (Fig. 2C). Moreover, overexpression of BCAR3 in FaDu cells accelerated cell growth (Fig. 2C). We further evaluated the effects of BCAR3 silencing using in vivo models. In accordance with the in vitro results, the subcutaneous xenografts of SCC25 cells in which BCAR3 was knocked down exhibited reduced growth and decreased tumor weight compared with those of control HNSCC cells, which indicates that interference in BCAR3 expression suppresses HNSCC growth in vivo. We also performed transwell assays to evaluate the effect of BCAR3 silencing on migration or invasion. The results showed that there were no significant differences between the groups (Supplementary Fig. 3).

### GO and KEGG analysis of BCAR3-related mRNAs

**Fig. 3:**
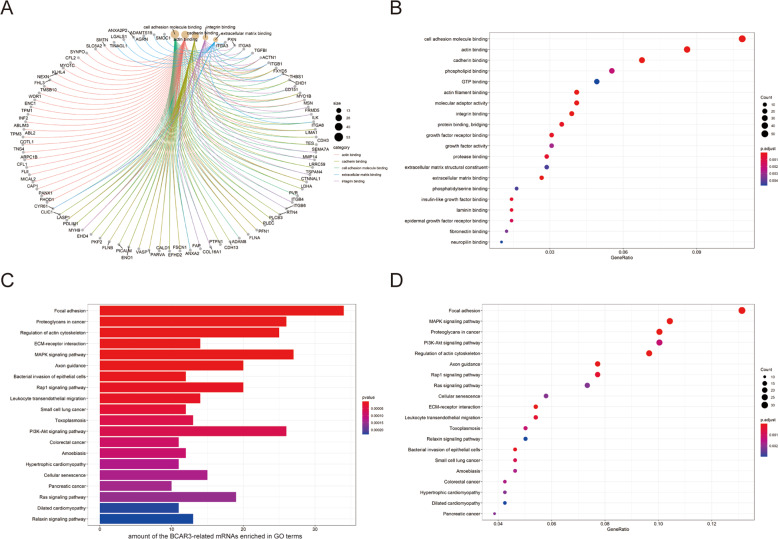
GO and KEGG pathway enrichment analyses of BCAR3-related mRNAs based on TCGA data. **A**, **B** Plots of the enriched GO terms in the GO enrichment analysis for BCAR3-related mRNAs. **C**, **D** Plots of the KEGG pathways in the KEGG pathway enrichment analysis for BCAR3-related mRNAs. *p* < 0.05 was used as the threshold to select GO and KEGG terms. GO, Gene Ontology; KEGG, Kyoto Encyclopedia of Genes and Genomes.

BCAR3-related mRNAs (Pearson’s correlation coefficient > 0.3 in the TCGA database) were selected for GO and KEGG pathway analyses to further explore the biological functions of BCAR3. A total of 48 GO terms and 37 KEGG pathways (*p* < 0.05) were identified, as shown in Fig. 3 and Supplementary Tables 1 and 2. The most significantly enriched GO terms for BCAR3-related mRNAs were cell adhesion molecule binding, actin binding, cadherin binding, and phospholipid binding, as shown in Fig. 3A, B. Similarly, the significant pathways for BCAR3-related mRNAs were mainly enriched in focal adhesion, the mitogen-activated protein kinase signaling pathway, proteoglycans in cancer, and the PI3K-Akt signaling pathway, as shown in Fig. 3C, D.

### GSEA identified BCAR3-related gene sets

**Fig. 4:**
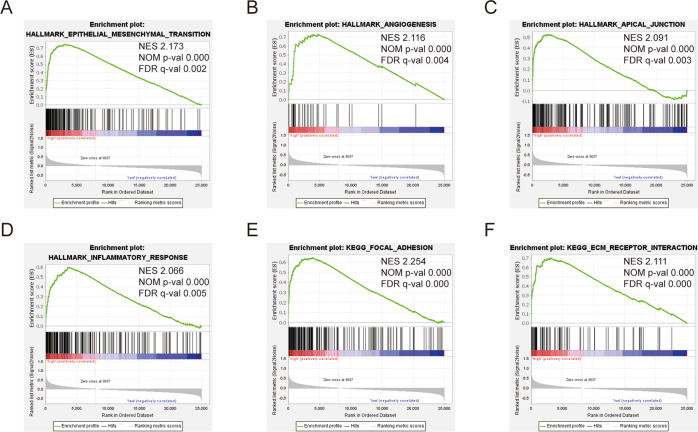
Enrichment plots from the GSEA. GSEA results show that “HALLMARK_EPITHELIAL_MESENCHYMAL_TRANSITION” (**A**), “HALLMARK_ANGIOGENESIS” (**B**), “HALLMARK_APICAL_JUNCTION” (**C**), “HALLMARK_INFLAMMATORY_RESPONSE” (**D**), “KEGG_FOCAL_ADHESION” (**E**), and “KEGG_ECM_RECEPTOR_INTERACTION” (**F**) were differentially enriched in HNSCC patients with high BCAR3 expression. NES, normalized enrichment score; NOM p-val, normalized *p*-value.

To identify BCAR3-associated features in HNSCC, we conducted GSEA between high and low BCAR3 expression data sets. GSEA revealed significant differences (FDR < 0.25, nominal *p*-value < 0.05) in the enrichment of “HALLMARK_EPITHELIAL_MESENCHYMAL_TRANSITION,” “HALLMARK_ANGIOGENESIS,” “HALLMARK_APICAL_JUNCTION,” “HALLMARK_INFLAMMATORY_RESPONSE,” “KEGG_FOCAL_ADHESION,” and “KEGG_ECM_RECEPTOR_INTERACTION” (h.all.v7.4; c2.cp.kegg.v7.4.symbols), which were prominently enriched in cases with high BCAR3 expression (Fig. 4). Other gene sets associated with high BCAR3 expression are shown in Table 3.

**Table 3:** Gene sets enriched in high BCAR3 samples.

MSigDB collection	Gene set name	NES	NOM *p*-val	FDR q-val
h.all.v7.4.symbols[Hallmarks]	HALLMARK_EPITHELIAL_MESENCHYMAL_TRANSITION	2.173	<0.001	0.002
	HALLMARK_ANGIOGENESIS	2.116	<0.001	0.004
	HALLMARK_APICAL_JUNCTION	2.091	<0.001	0.003
	HALLMARK_INFLAMMATORY_RESPONSE	2.066	<0.001	0.005
	HALLMARK_COMPLEMENT	2.033	<0.001	0.005
	HALLMARK_TGF_BETA_SIGNALING	2.025	<0.001	0.004
	HALLMARK_TNFA_SIGNALING_VIA_NFKB	2.016	0.002	0.005
	HALLMARK_COAGULATION	1.997	0.004	0.005
	HALLMARK_IL6_JAK_STAT3_SIGNALING	1.915	0.004	0.011
	HALLMARK_APOPTOSIS	1.907	0.002	0.011
	HALLMARK_HYPOXIA	1.830	0.012	0.020
	HALLMARK_INTERFERON_GAMMA_RESPONSE	1.829	0.020	0.018
	HALLMARK_KRAS_SIGNALING_UP	1.822	0.004	0.018
c2.cp.kegg.v7.4.symbols[Curated]	KEGG_FOCAL_ADHESION	2.254	<0.001	<0.001
	KEGG_ECM_RECEPTOR_INTERACTION	2.111	<0.001	<0.001
	KEGG_LEUKOCYTE_TRANSENDOTHELIAL_MIGRATION	1.955	0.020	0.027
	KEGG_REGULATION_OF_ACTIN_CYTOSKELETON	1.926	<0.001	0.024

Gene sets with NES > 1.9 were listed.

*FDR* false discovery rate, *NES* normalized enrichment score, *NOM* nominal.

## Discussion

BCAR3, a member of the novel Src homology 2 (SH2)-containing protein (NSP) family, was first identified in the search for genes involved in the development of estrogen resistance in breast cancer [5]. This protein contains an NH2-terminal SH2 domain and a COOH-terminal guanine nucleotide exchange factor-like domain with homology to the CDC25 family [6, 10]. Through its COOH-terminal domain, BCAR3 binds to p130^Cas^ (BCAR1) [11] and functions in promoting p130^Cas^ membrane localization and membrane ruffling [7], activating small GTPases, including Rap1, R-Ras, RalA, Cdc42, and Rac1 [12, 13], regulating Src/p130^Cas^ association and Src kinase activity [10, 14], inhibiting transforming growth factor (TGF)-β/Smad signaling [15], and promoting adhesion disassembly and cellular invasion [16].

The role of BCAR3 in cancer is mainly reported in breast cancer. The majority of studies at the cell level found that BCAR3 promotes breast cancer progression through the development of estrogen resistance [5], promotion of CCND1 expression [6], promotion of migration and invasion [7, 16], and regulation of adhesion signaling [10]. However, clinical observations found that a high level of BCAR3 mRNA is significantly associated with favorable survival [8, 9]. Lebrun and colleagues [15] indicated that BCAR3 acts as a putative suppressor of breast cancer progression by inhibiting the prometastatic transforming growth TGFβ/Smad signaling pathway, which helps explain the differences among studies. Studies on BCAR3 in other cancers are quite scarce and the only study in solid tumors showed that BCAR3 inhibition suppresses ovarian cancer cell proliferation [17]. It was also found that a high expression level of BCAR3 predicts a better prognosis in multiple myeloma patients [18].

The role of BCAR3 in HNSCC remains unclear and, thus, in the present study, we explored the possible role of BCAR3 in HNSCC. We found that BCAR3 was overexpressed in tumor tissues. Notably, this genetic alteration had a small fold change and should be considered carefully. Nonetheless, this result merits further validation. Survival analyses demonstrated that BCAR3 was significantly associated with a poor prognosis. We found that BCAR3 promoted HNSCC cell proliferation and tumor growth in vivo, which could partially account for the observed decreased survival. In addition, it was found that BCAR3 expression was associated with perineural invasion, an important process of cancer dissemination. Perineural invasion was found to be an adverse factor in HNSCC that greatly worsens patient survival, as reported in our previous study and in other studies [19, 20]. However, establishing reliable models that can be used to evaluate perineural invasion is relatively difficult; thus, we were unable to explore the effect of BCAR3 on perineural invasion, which is a limitation of the present study. The relationship between BCAR3 and perineural invasion will be investigated in our future work.

The present study showed that knockdown of BCAR3 in HNSCC reduces the proliferative capacity of HNSCC in vitro and in vivo. Notably, previous studies showed that BCAR3 acts as a regulator in EGF- or insulin-induced DNA synthesis. Moreover, it was found that BCAR3 activates the CCND1 promoter and CCND1 is believed to be an important tumor promotor in HNSCC that drives cells through the G1–S checkpoint of the cell cycle and contributes to unscheduled DNA replication [21]. These findings may explain why BCAR3 inhibition suppresses HNSCC cell proliferation.

In the past, studies have primarily focused on the functions of specific individual genes. As high-throughput technologies have advanced, bioinformatics analyses have been widely applied to reveal many molecular changes in cancer. The biological processes in which BCAR3 may be involved in have not been well investigated. Here we identified BCAR3-related mRNAs by calculating the Pearson’s correlation coefficients. GO and KEGG functional enrichment analyses of BCAR3-related mRNAs suggested that BCAR3 expression in HNSCC was associated with biological processes, such as cell adhesion molecule binding, actin binding, and cadherin binding. These findings suggest that the role of BCAR3 in these biological processes or phenotypes in HNSCC, which remains largely unknown, deserves to be evaluated in further studies. GSEA showed that BCAR3 likely participates in the processes of EMT, angiogenesis, apical junction, focal adhesion, and extracellular matrix and receptor interaction, as genes associated with these biological processes are overexpressed in the high-BCAR3 cases. Most of the enriched pathways or biological processes are involved in regulating cancer invasion or metastasis. Moreover, it was reported that BCAR3 promotes cell motility, focal adhesions, and invasive capacity in breast cancer cells [16, 22, 23]. We speculate that BCAR3 also promotes the migration and invasiveness of HNSCC; thus, we performed Transwell assays to evaluate the effect of BCAR3 expression on the migration and invasiveness of HNSCC. However, we found no significant difference between BCAR3-deficient cells and control cells. This inconsistency could be due to different cellular environments. We hypothesize that BCAR3 may function more in promoting the interaction between cancer cells and nerve cells to promote perineural invasion rather than simply promoting cell migration. However, this speculation requires more experimental data to support in further study. The relationship between BCAR3 and migration or invasiveness of HNSCC should be further investigated.

## Conclusions

In summary, BCAR3 expression is associated with the presence of perineural invasion and may be a potential molecular prognostic marker of poor survival in HNSCC. BCAR3 promotes the proliferation of HNSCC cells in vitro and tumor growth in vivo. Therefore, BCAR3 might be a new therapeutic target and may serve as a means for preventing HNSCC.

## Supplementary information

Supplementary Figure Legends

SUPPLEMENTAL MATERIAL figure S1

SUPPLEMENTAL MATERIAL figure S2

SUPPLEMENTAL MATERIAL figure S3

SUPPLEMENTAL MATERIAL Table 1

SUPPLEMENTAL MATERIAL Table 2

## Data Availability

The data sets generated during and/or analyzed during the current study are available from the corresponding author on reasonable request.
