# Supplementary material for: BCAR3 promotes head and neck cancer growth and is associated with poor prognosis
Source: Cell Death Discov. 2021 Oct 27;7:316. doi: 10.1038/s41420-021-00714-7 (PMC8551282; doi:10.1038/s41420-021-00714-7)
Supplement: Supplementary file 1 — Supplementary Figure Legends [file 41420_2021_714_MOESM1_ESM.docx]

Supplementary Figure 1 BCAR3 is upregulated in HNSCC. (A) The expression level of BCAR3 was upregulated in oral cancer tissues (n=22) compared with normal tissues (n=24) in a GEO cohort (GSE31056).

Supplementary Figure 2 Silencing BCAR3 in SCC25 and FaDu cells by two individual shRNAs significantly decreased BCAR3 expression, as detected by western blotting.

Supplementary Figure 3 Migration or invasion of silenced BRAC3 cells was evaluated by transwell assays. ‘control’ represents ‘scrambled siRNA’.
