# Supplementary figures and images for: BCAR3 promotes head and neck cancer growth and is associated with poor prognosis
Source: Cell Death Discov. 2021 Oct 27;7:316. doi: 10.1038/s41420-021-00714-7 (PMC8551282; doi:10.1038/s41420-021-00714-7)

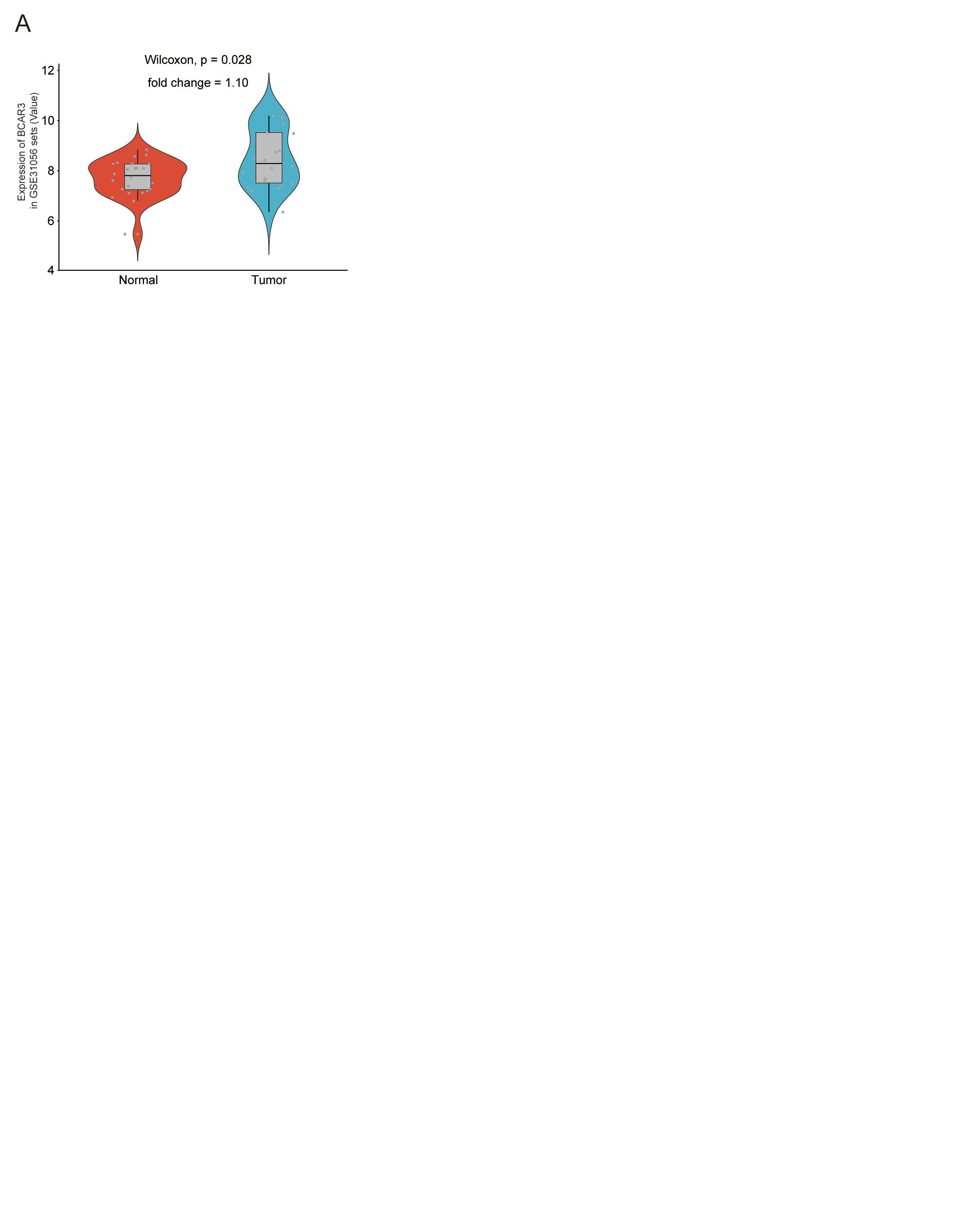

Supplement: Supplementary file 2 — SUPPLEMENTAL MATERIAL figure S1 [file 41420_2021_714_MOESM2_ESM.png]

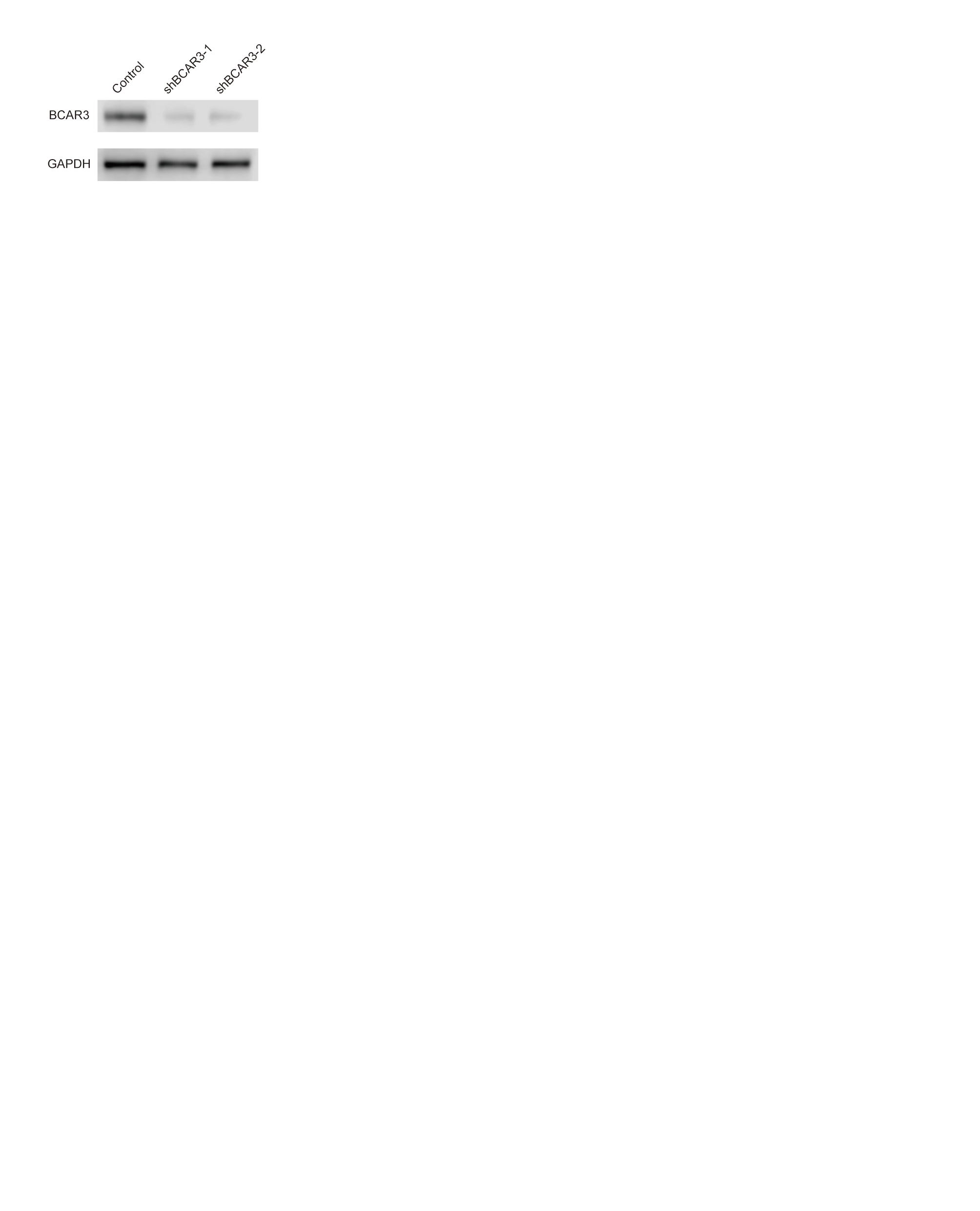

Supplement: Supplementary file 3 — SUPPLEMENTAL MATERIAL figure S2 [file 41420_2021_714_MOESM3_ESM.png]

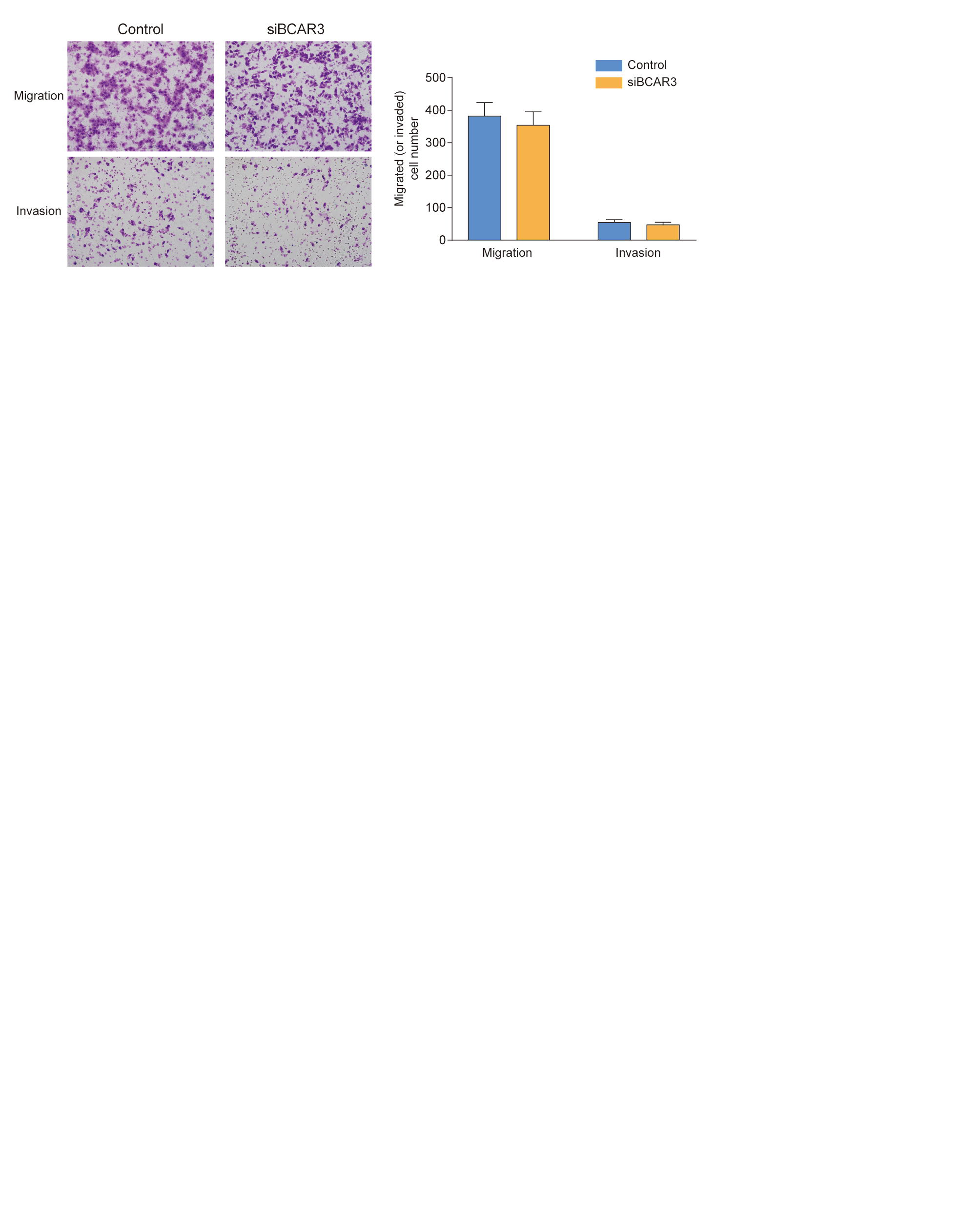

Supplement: Supplementary file 4 — SUPPLEMENTAL MATERIAL figure S3 [file 41420_2021_714_MOESM4_ESM.png]
