# Supplementary material for: BCAR3 promotes head and neck cancer growth and is associated with poor prognosis
Source: Cell Death Discov. 2021 Oct 27;7:316. doi: 10.1038/s41420-021-00714-7 (PMC8551282; doi:10.1038/s41420-021-00714-7)
Supplement: Supplementary file 5 — SUPPLEMENTAL MATERIAL Table 1 [file 41420_2021_714_MOESM5_ESM.pdf]

ID	Description	GeneRatio
GO:0050839	cell adhesion molecule binding	53/488
GO:0003779	actin binding	42/488
GO:0045296	cadherin binding	33/488
GO:0005178	integrin binding	19/488
GO:0050840	extracellular matrix binding	13/488
GO:0051015	actin filament binding	20/488
GO:0060090	molecular adaptor activity	20/488
GO:0070851	growth factor receptor binding	15/488
GO:0030674	protein binding, bridging	17/488
GO:0005520	insulin-like growth factor binding	7/488
GO:0002020	protease binding	14/488
GO:0043236	laminin binding	7/488
GO:0005154	epidermal growth factor receptor binding	7/488
GO:0005543	phospholipid binding	27/488
GO:0008083	growth factor activity	15/488
GO:0001968	fibronectin binding	6/488
GO:0005201	extracellular matrix structural constituent	14/488
GO:0001786	phosphatidylserine binding	8/488
GO:0005525	GTP binding	24/488
GO:0038191	neuropilin binding	5/488
GO:0032550	purine ribonucleoside binding	24/488
GO:0048018	receptor ligand activity	28/488
GO:0001883	purine nucleoside binding	24/488
GO:0032549	ribonucleoside binding	24/488
GO:0004859	phospholipase inhibitor activity	4/488
GO:0001882	nucleoside binding	24/488
GO:0003924	GTPase activity	20/488
GO:0019001	guanyl nucleotide binding	24/488
GO:0032561	guanyl ribonucleotide binding	24/488
GO:0004222	metalloendopeptidase activity	11/488
GO:0019838	growth factor binding	12/488
GO:0005539	glycosaminoglycan binding	16/488
GO:0005160	transforming growth factor beta receptor bindir	7/488
GO:0005518	collagen binding	8/488
GO:0015293	symporter activity	12/488
GO:1902936	phosphatidylinositol bisphosphate binding	10/488
GO:0072341	modified amino acid binding	9/488
GO:0005546	phosphatidylinositol-4,5-bisphosphate binding	8/488
GO:0045499	chemorepellent activity	5/488
GO:0055102	lipase inhibitor activity	4/488
GO:0098631	cell adhesion mediator activity	7/488
GO:1901981	phosphatidylinositol phosphate binding	12/488
GO:0046332	SMAD binding	8/488
GO:0098632	cell-cell adhesion mediator activity	6/488
GO:0050431	transforming growth factor beta binding	4/488
GO:0035091	phosphatidylinositol binding	15/488
GO:0005544	calcium-dependent phospholipid binding	6/488
GO:0030215	semaphorin receptor binding	4/488

BgRatio	pvalue	p.adjust	qvalue
486/17632	1.01E-17	7.13E-15	5.87E-15
421/17632	6.25E-13	2.20E-10	1.81E-10
323/17632	1.09E-10	2.56E-08	2.11E-08
126/17632	1.83E-09	3.22E-07	2.65E-07
56/17632	3.04E-09	4.28E-07	3.53E-07
190/17632	3.34E-07	3.92E-05	3.23E-05
196/17632	5.52E-07	5.55E-05	4.58E-05
132/17632	3.82E-06	0.000336476	0.00027721
168/17632	4.37E-06	0.000341494	0.000281344
28/17632	8.52E-06	0.000599602	0.000493991
125/17632	9.50E-06	0.000607716	0.000500675
29/17632	1.10E-05	0.000643043	0.00052978
33/17632	2.73E-05	0.001476196	0.001216185
417/17632	4.16E-05	0.002093382	0.001724661
166/17632	6.00E-05	0.002818054	0.002321692
27/17632	7.86E-05	0.003460517	0.002850994
155/17632	0.000105506	0.00432151	0.003560335
54/17632	0.000110493	0.00432151	0.003560335
375/17632	0.000131695	0.004721689	0.003890028
19/17632	0.000134139	0.004721689	0.003890028
380/17632	0.000160897	0.005393875	0.004443818
478/17632	0.000168624	0.005395983	0.004445555
383/17632	0.000181057	0.005522241	0.004549574
384/17632	0.000188258	0.005522241	0.004549574
12/17632	0.000240411	0.00666581	0.005491719
391/17632	0.00024618	0.00666581	0.005491719
301/17632	0.000289682	0.00700921	0.005774633
396/17632	0.000296689	0.00700921	0.005774633
396/17632	0.000296689	0.00700921	0.005774633
113/17632	0.000298688	0.00700921	0.005774633
137/17632	0.000429106	0.009744867	0.008028442
222/17632	0.000476465	0.010257707	0.008450952
51/17632	0.00048083	0.010257707	0.008450952
67/17632	0.000506173	0.010480759	0.008634716
142/17632	0.000593359	0.011716879	0.009653111
104/17632	0.000614112	0.011716879	0.009653111
86/17632	0.000615802	0.011716879	0.009653111
71/17632	0.000749916	0.013893189	0.011446093
27/17632	0.0007758	0.014004192	0.011537544
17/17632	0.001035479	0.018224432	0.015014447
59/17632	0.001171891	0.020122226	0.01657797
154/17632	0.001212421	0.020322481	0.016742953
80/17632	0.001646345	0.026954106	0.022206508
50/17632	0.002468128	0.039490051	0.032534417
22/17632	0.002852497	0.044435753	0.036609001
240/17632	0.002903473	0.044435753	0.036609001
53/17632	0.003326271	0.049550531	0.040822881
23/17632	0.003378445	0.049550531	0.040822881

geneID	Count
ITGA3/PXN/ITGA5/TGFBI/ACTN1/ITGB1/FXYD5/T	53
ACTN1/ITGB1/FHOD1/FXYD5/PANX1/CAP1/MIC/	42
ITGB1/FXYD5/EHD1/MYO1B/ITGA6/LIMA1/CDH3	33
ITGA3/PXN/ITGA5/TGFBI/ACTN1/ITGB1/THBS1/C	19
ITGA3/TGFBI/ITGB1/THBS1/TINAGL1/LGALS1/ITG	13
ACTN1/PANX1/MYO1B/LIMA1/FLII/CFL1/ARPC1I	20
CAV1/ANXA5/CAV2/SLC9A1/SHC1/AP2B1/IRS1/	20
VEGFC/ITGA5/CSF2/AREG/VAV2/FGF5/APP/SHC	15
CAV1/CAV2/SLC9A1/SHC1/AP2B1/IRS1/FSCN1/E	17
ITGA6/ITGB4/CRIM1/HTRA1/IGFBP7/IGFBP6/CYR	7
ITGA3/SERPINE1/F3/ITGB1/CCBE1/PANX1/PRNP/	14
ITGA3/ITGB1/THBS1/TINAGL1/LGALS1/ITGA6/AG	7
ITGA5/AREG/VAV2/SHC1/TGFA/EFEMP1/EREG	7
F3/PLEK2/ANXA5/THBS1/MYO1B/AXL/JPH2/SLC	27
VEGFC/INHBA/CSF2/AREG/FGF5/IL11/BMP1/PDG	15
ITGA3/FSTL3/ITGB1/THBS1/SDC4/IGFBP6	6
LAMC2/LAMA3/TGFBI/THBS1/TINAGL1/COL17A1	14
THBS1/AXL/JPH2/ANXA2P2/ANXA2/GAS6/SYT7/	8
EHD1/EHD2/TUBB6/RRAS/RRAS2/RAB31/SCG5/F	24
PXN/SEMA3C/SEMA7A/SEMA3A/SEMA3B	5
EHD1/EHD2/TUBB6/RRAS/RRAS2/RAB31/SCG5/F	24
VEGFC/INHBA/WNT7A/CSF2/AREG/SEMA3C/FGF	28
EHD1/EHD2/TUBB6/RRAS/RRAS2/RAB31/SCG5/F	24
EHD1/EHD2/TUBB6/RRAS/RRAS2/RAB31/SCG5/F	24
ANXA5/ANXA2P2/ANXA3/ANXA2	4
EHD1/EHD2/TUBB6/RRAS/RRAS2/RAB31/SCG5/F	24
TUBB6/RRAS/RRAS2/RAB31/RHOB/RHOC/RAP1A	20
EHD1/EHD2/TUBB6/RRAS/RRAS2/RAB31/SCG5/F	24
EHD1/EHD2/TUBB6/RRAS/RRAS2/RAB31/SCG5/F	24
TLL1/MMP28/MMP10/MMP14/ADAMTS6/BMP1/	11
THBS1/SHC1/ITGA6/TGFBR2/ITGB4/LTBP1/CRIM1	12
LAMC2/ANXA5/THBS1/APP/PRNP/TGFBR2/NLRP	16
INHBA/TGFBR2/BMP2/TGFB1/SMURF2/TGFBR1/T	7
ITGA3/TGFBI/ITGB1/CCBE1/THBS1/SERPINH1/CD	8
SLC12A4/SLC20A1/SLC16A2/SLC22A3/SLC22A4/	12
PLEK2/MYO1B/JPH2/SLC9A1/ANXA2P2/PFN1/AN	10
THBS1/AXL/JPH2/ANXA2P2/ANXA2/FOLR3/GAS	9
MYO1B/JPH2/SLC9A1/ANXA2P2/PFN1/ANXA2/P	8
SEMA3C/SEMA7A/FLRT2/SEMA3A/SEMA3B	5
ANXA5/ANXA2P2/ANXA3/ANXA2	4
ITGB1/DSCAM/ANXA2/PKP2/NEXN/PDLIM1/SIRF	7
PLEK2/MYO1B/JPH2/SLC9A1/ANXA2P2/PFN1/AN	12
TGFBR2/HMGA2/FLNA/PMEPA1/BMP2/SMURF2/	8
DSCAM/ANXA2/PKP2/NEXN/PDLIM1/SIRPA	6
THBS1/TGFBR2/LTBP1/TGFBR1	4
PLEK2/MYO1B/JPH2/SLC9A1/ANXA2P2/PFN1/AN	15
ANXA5/ANXA2P2/ANXA3/ANXA2/ESYT1/SYT7	6
SEMA3C/SEMA7A/SEMA3A/SEMA3B	4
