# Supplementary material for: BCAR3 promotes head and neck cancer growth and is associated with poor prognosis
Source: Cell Death Discov. 2021 Oct 27;7:316. doi: 10.1038/s41420-021-00714-7 (PMC8551282; doi:10.1038/s41420-021-00714-7)
Supplement: Supplementary file 6 — SUPPLEMENTAL MATERIAL Table 2 [file 41420_2021_714_MOESM6_ESM.pdf]

ID	Description
hsa04510	Focal adhesion
hsa05205	Proteoglycans in cancer
hsa04810	Regulation of actin cytoskeleton
hsa04512	ECM-receptor interaction
hsa04010	MAPK signaling pathway
hsa04360	Axon guidance
hsa05100	Bacterial invasion of epithelial cells
hsa04015	Rap1 signaling pathway
hsa04670	Leukocyte transendothelial migration
hsa05222	Small cell lung cancer
hsa05145	Toxoplasmosis
hsa04151	PI3K-Akt signaling pathway
hsa05210	Colorectal cancer
hsa05146	Amoebiasis
hsa05410	Hypertrophic cardiomyopathy
hsa04218	Cellular senescence
hsa05212	Pancreatic cancer
hsa04014	Ras signaling pathway
hsa05414	Dilated cardiomyopathy
hsa04926	Relaxin signaling pathway
hsa04933	AGE-RAGE signaling pathway in diabetic complic
hsa05132	Salmonella infection
hsa05220	Chronic myeloid leukemia
hsa04350	TGF-beta signaling pathway
hsa05131	Shigellosis
hsa05142	Chagas disease
hsa04520	Adherens junction
hsa05225	Hepatocellular carcinoma
hsa05133	Pertussis
hsa04144	Endocytosis
hsa05412	Arrhythmogenic right ventricular cardiomyopathy
hsa00532	Glycosaminoglycan biosynthesis - chondroitin su
hsa04640	Hematopoietic cell lineage
hsa04371	Apelin signaling pathway
hsa05135	Yersinia infection
hsa04390	Hippo signaling pathway
hsa05211	Renal cell carcinoma

GeneRatio	BgRatio	pvalue	p.adjust
34/259	201/8105	5.57E-16	1.44E-13
26/259	205/8105	1.41E-09	1.82E-07
25/259	218/8105	2.38E-08	2.04E-06
14/259	88/8105	6.33E-07	3.38E-05
27/259	294/8105	6.54E-07	3.38E-05
20/259	182/8105	1.27E-06	5.45E-05
12/259	77/8105	5.08E-06	0.000187395
20/259	210/8105	1.15E-05	0.000371699
14/259	114/8105	1.46E-05	0.000419301
12/259	92/8105	3.28E-05	0.000845893
13/259	112/8105	5.42E-05	0.001270347
26/259	354/8105	5.94E-05	0.001277901
11/259	86/8105	8.39E-05	0.001665792
12/259	102/8105	9.19E-05	0.001694364
11/259	90/8105	0.00012751	0.002106517
15/259	156/8105	0.000131508	0.002106517
10/259	76/8105	0.000138802	0.002106517
19/259	232/8105	0.000150851	0.002162193
11/259	96/8105	0.000228227	0.002986608
13/259	129/8105	0.00023152	0.002986608
11/259	100/8105	0.000327483	0.004023363
19/259	249/8105	0.000374377	0.00439042
9/259	76/8105	0.00066245	0.007430958
10/259	94/8105	0.000794344	0.0085392
18/259	246/8105	0.000873326	0.009012727
10/259	102/8105	0.001497008	0.014854924
8/259	71/8105	0.001818322	0.017375078
13/259	168/8105	0.002761174	0.024784378
8/259	76/8105	0.002810395	0.024784378
17/259	252/8105	0.002881904	0.024784378
8/259	77/8105	0.003051647	0.025397579
4/259	20/8105	0.003294763	0.026564029
9/259	99/8105	0.004261046	0.031453079
11/259	137/8105	0.00433713	0.031453079
11/259	137/8105	0.00433713	0.031453079
12/259	157/8105	0.004388802	0.031453079
7/259	69/8105	0.006251132	0.043588978

qvalue	geneID	Count
1.13E-13	3675/5829/857/7424/3678/3914	34
1.42E-07	5829/857/3678/3688/5328/858/	26
1.60E-06	3675/5829/3678/87/3688/9564/	25
2.64E-05	3675/3678/3914/3918/3909/368	14
2.64E-05	7424/6195/374/1848/2250/6237	27
4.27E-05	3688/10512/6237/3897/3611/19	20
0.000146796	5829/857/3678/3688/858/9564/	12
0.000291172	7424/3688/7057/9564/60/7410/	20
0.000328461	5829/87/3688/9564/4478/60/74	14
0.000662633	3675/3914/3918/3909/3688/365	12
0.000995131	3914/3918/3909/3688/3655/704	13
0.001001049	3675/7424/3678/3914/3918/390	26
0.001304904	374/7048/5293/578/7039/5880/	11
0.001327286	3914/3918/3909/87/1437/5331/	12
0.001650148	3675/3678/3688/60/3655/3691/	11
0.001650148	5054/2113/6237/22800/7048/10	15
0.001650148	7048/1021/5293/578/7039/5880	10
0.001693762	7424/2113/2250/6237/6464/336	19
0.002339571	3675/3678/3688/60/3655/3691/	11
0.002339571	7424/6464/7048/5331/5293/704	13
0.003151716	7424/5054/2152/7048/5331/355	11
0.003439252	29109/84617/60/6237/10095/38	19
0.005821069	6464/7048/1021/5293/578/7040	9
0.006689214	3624/7057/7048/650/7040/6475	10
0.007060154	5829/3678/87/3688/9564/1437/	18
0.01163666	5054/7048/5331/5293/7040/709	10
0.013610832	87/6591/60/7048/5770/5880/42	8
0.019414935	7476/60/6464/7048/1021/5293/	13
0.019414935	3678/3688/1072/3552/114548/1	8
0.019414935	857/858/10938/30846/163/1103	17
0.019895288	3675/3678/3688/60/3655/3691/	8
0.020809032	29940/50515/51363/55454	4
0.024638887	3675/3678/1437/966/3655/3589	9
0.024638887	5054/6237/6548/22800/5331/88	11
0.024638887	5829/3678/3688/9564/6195/60/	11
0.024638887	5054/7476/6591/60/374/7048/6	12
0.034145589	2113/5293/5906/7039/4233/704	7
